# Role of Early Assesment of Diuresis and Natriuresis in Detecting In-Hospital Diuretic Resistance in Acute Heart Failure

**DOI:** 10.3389/fphys.2022.887734

**Published:** 2022-05-02

**Authors:** Belén García-Magallón, Marta Cobo-Marcos, Aitor Dávila Martiarena, Esther Montero Hernández, Maria Luisa Martín Jiménez, Aránzazu Martín García, Daniel De Castro Campos, Paula Vela Martín, Fernando Hernández Terciado, Ramón Garrido González, Andrea Matutano Muñoz, Daniel Escribano García, Fernando Domínguez, Ana Sainz Herrero, Camino Gómez Peñalba, Pablo Garcia-Pavia, Javier Segovia

**Affiliations:** ^1^ Department of Cardiology, Hospital Universitario Puerta de Hierro Majadahonda, IDIPHISA, Madrid, Spain; ^2^ Centro de Investigación Biomédica en Red en Enfermedades Cardiovasculares (CIBERCV), Madrid, Spain; ^3^ Emergency Department, Hospital Universitario Puerta de Hierro Majadahonda, IDIPHISA, Madrid, Spain; ^4^ Department of Internal Medicine, Hospital Universitario Puerta de Hierro Majadahonda, IDIPHISA, Madrid, Spain; ^5^ Department of Laboratory of Biochemistry-Clinical Analysis, Hospital Universitario Puerta de Hierro Majadahonda, IDIPHISA, Madrid, Spain; ^6^ Universidad Francisco de Vitoria (UFV), Madrid, Spain

**Keywords:** diuretic, acute heart failure, natriuresis, diuretic response, diuretic resistance

## Abstract

**Background and Purpose:** European Guidelines recommend early evaluation of diuresis and natriuresis after the first administration of diuretic to identify patients with insufficient diuretic response during acute heart failure. The aim of this work is to evaluate the prevalence and characteristics of patients with insufficient diuretic response according to this new algorithm.

**Methods:** Prospective observational single centre study of consecutive patients with acute heart failure and congestive signs. Clinical evaluation, echocardiography and blood tests were performed. Diuretic naïve patients received 40 mg of intravenous furosemide. Patients on an oupatient diuretic regimen received 2 times the ambulatory dose. The diuresis volume was assessed 6 h after the first loop diuretic administration, and a spot urinary sample was taken after 2 h. Insufficient diuretic response was defined as natriuresis <70 mEq/L or diuresis volume <600 ml.

**Results:** From January 2020 to December 2021, 73 patients were included (59% males, median age 76 years). Of these, 21 patients (28.8%, 95%CI 18.4; 39.2) had an insufficient diuretic response. Diuresis volume was <600 ml in 13 patients (18.1%), and 12 patients (16.4%) had urinary sodium <70 mEq/L. These patients had lower systolic blood pressure, worse glomerular filtration rate, and higher aldosterone levels. Ambulatory furosemide dose was also higher. These patients required more frequently thiazides and inotropes during admission.

**Conclusion:** The diagnostic algorithm based on diuresis and natriuresis was able to detect up to 29% of patients with insufficient diuretic response, who showed some characteristics of more advanced disease.

## Introduction

Signs and symptoms of congestion are usually the most common manifestations among patients with acute heart failure (HF) (Adams et al., 2005), and intravenous loop diuretics remain the most widely used therapy to achieve euvolaemia (Fonarow et al., 2004). Diuretic response is defined as the capacity of diuretics to induce natriuresis and diuresis (ter Maaten et al., 2015a).

Identification of patients who may have a poor diuretic response is one of the most important challenges in the field of HF, since a poor diuretic response is associated with a higher risk of rehospitalization and increased mortality (Metra et al., 2012; Neuberg et al., 2002; Valente et al., 2014; ter Maaten et al., 2015b; Testani et al., 2014; Voors et al., 2014). To date, no uniform and standard definition was available to allow the early identification of patients at risk of developing resistance to diuretic treatment during HF hospitalization.

The Position Statement from the Heart Failure Association of the European Society of Cardiology about the use of diuretics in heart failure with congestion (Mullens et al., 2019), and more recently the European Guidelines for the diagnosis and treatment of acute and chronic heart failure (McDonagh et al., 2021), have proposed an algorithm that includes the early assessment of diuresis and natriuresis after the first administration of loop diuretics in patients with acute HF, in order to detect patients with insufficient diuretic response who might benefit from diuretic intensification.

To date, data on the prevalence of early diuretic resistance according to these parameters have not yet been described.

The aim of this work is to evaluate the prevalence and features of acute HF patients who present an insufficient diuretic response according to this algorithm.

## Methods

From January 2020 to December 2021, we conducted a prospective, observational and single centre study on a sample of consecutive patients aged ≥18 years whose primary admission diagnosis was acute HF and were admitted to the cardiology department. The diagnosis of acute HF was based on the current ESCF HF guidelines. In addition, NTproBNP >300 pg/dl and the presence of at least two of the following congestion criteria were required: jugular venous pressure >10 cm, lower limb edema, ascites, or pleural effusion determined by chest x-ray or pulmonary ultrasound.

Patients in cardiogenic shock and/or on dialysis were excluded. Patients in whom urine output or natriuresis could not be recorded or were missed were also excluded.

### Study Procedures and Statistical Analysis

Complete clinical evaluation, echocardiogram and laboratory tests were performed. Diuretic naïve patients received 40 mg of intravenous furosemide. Patients on an outpatient diuretic regimen received 2 times the home dose. The diuresis volume was assessed 6 h after the first loop diuretic administration, and a spot urinary sample was taken after 2 h. Urinary sodium was measured using a Siemens Dimension EXL chemistry analyzer. Insufficient diuretic response was defined as natriuresis <70 mEq/L or diuresis volume <600 ml.

Values of continuous variables are given as the median and interquartile range (IQR). Categorical variables are described in absolute and relative frequencies. The associations between clinical characteristics and diuretic response were analyzed by univariate analysis using the Chi square test for categorical variables and the Mann-Whitney U test for contiuous variables. A *p*-value <0.05 was considered significant. All analyses were performed using STATA v.13 (StataCorp. 2013. Stata Statistical Software: Release 13. College Station, TX) and R software (R Foundation for Statistical Computing, version 3.6.0).

The present study conforms to the principles of the Declaration of Helsinki. Approval from the local ethics committee/internal review board was obtained at the participating centers and patients signed an informed consent.

## Results

From January 2020 to December 2021, 694 patients were admitted for acute HF. Nearly 50% of these patients did not meet the inclusion criteria as they presented predominant pulmonary congestion. About 30% could not be included as the treating physician didn’t follow the ESC protocol, in part due to Covid-19 pandemic.

A final sample of 73 patients were included (59% males, median age 76 years [IQR: 70–85]). Four initially included patients were not finally analysed as urinary output was not correctly collected. Of the remaining sample (73/78), 21 patients (28.8%) met the definition of early insufficient diuretic response.

The diuresis volume was <600 ml in 13 patients (18.1%), and 12 patients (16.4%) had urinary sodium <70 mEq/L. Only 4 patients (5.5%) had both low urinary sodium and decreased urine output.

Compared with patients with an adequate diuretic response, these patients had lower systolic blood pressure (133 mmHg [IQR: 116–148] vs. 148 [IQR: 124–175], *p* = 0.043), worse glomerular filtration rate (49 ml/min/1.73 m^2^ [IQR: 28–63] vs. 69 [44–86], *p* = 0.044), and showed greater neurohormonal activation (aldosterone levels: 19 ng/dl [IQR: 11–40] vs. 10 [IQR: 7–14], *p* = 0.005). This group of patients presented a higher percentage of previous admission due to HF (42.9 vs. 17.6%, *p* = 0.025), and their basal furosemide dose was also higher (80 mg [IQR: 5–88] vs. 40 [IQR: 0–60], *p* = 0.032) Table 1.

**TABLE 1:** Legend. Patient characteristics per diuretic response (*n* = 73).

Patient characteristics per diuretic response	All patients *n* = 73	Poor DR *n* = 21 (28.8%)	Good DR *n* = 52 (71.2%)	*p*-value
Age (years)^a^	76 (70–85)	76 (50–87)	78 (70–85)	0.845
Male *n* (%)	43 (58.9)	12 (57.1)	31 (59.6)	0.846
Diabetes mellitus 2, *n* (%)	29 (39.7)	12 (57.1)	17 (32.7)	0.137
Arterial hypertension, *n* (%)	62 (84.9)	18 (85.7)	44 (84.6)	0.905
Chronic kidney disease, *n* (%)	17 (23.3)	6 (28.6)	11 (21.1)	0.575
Atrial Fibrillation, *n* (%)	43 (58.9)	14 (66.7)	29 (56.9)	0.441
Previous hospitalization for heart failure, *n* (%)	18 (24.7)	9 (42.9)	9 (17.6)	**0.025**
De novo HF, *n* (%)	26 (35.6)	5 (23.8)	21 (40.4)	0.280
Heart failure evolution (days)^a^	293 (0–1965)	436 (0–3,030)	232 (0–1,647)	0.250
Basal treatment				
Furosemide, *n* (%)	48 (66)	15 (71)	33 (64)	0.594
Ambulatory furosemide dose^a^ (mg)	40 (0–80)	80 (5–88)	40 (0–60)	**0.032**
MRA, n (%)	8 (11)	2 (10)	6 (12)	0.583
Thiazides, *n* (%)	6 (8)	3 (14)	3 (6)	0.345
Acetazolamide,*n* (%)	2 (2.7)	1 (4.8)	0 (0)	0.080
SLGT2i, *n* (%)	3 (4)	1 (5)	2 (4)	0.645
RAASi, *n* (%)	41 (56)	10 (48)	31 (60)	0.427
Betablockers, *n* (%)	33 (45)	11 (52)	22 (42)	0.450
Admission				
Charlson score^a^	2 (1–3)	2 (2–4)	2 (1-3)	0.212
LVEF (%)^a^	55 (38–60)	55 (31–59)	55 (40-60)	0.225
Type of HF, *n* (%)	—	—	—	0.235
HFrEF	24 (33.8)	7 (33.3)	17 (32.7)	—
HFmrEF	9 (12.3)	2 (9.5)	7 (13.5)	—
HFpEF	40 (54.7)	12 (57.1)	28 (53.8)	—
TAPSE (mm)^a^	17 (14-21)	16 (14–21)	17 (14–21)	0.680
Tricuspid regurgitation III-IV, *n* (%)	10 (13.7)	2 (9.5)	8 (15.4)	0.714
sPAP (mmHg)^a^	45 (35–55)	50 (40–55)	45 (35–55)	0.491
Systolic blood pressure (mmHg)^a^	141 (123–166)	133 (116–148)	148 (124–175)	**0.043**
Diastolic blood pressure (mmHg)^a^	76 (69–86)	75 (65–83)	76 (69–90)	0.227
Everest score^a^	11 (9–12)	11 (9–13)	11 (8–12)	0.186
Inferior cava vein (mm)^a^	22 (19–24)	23 (20–26)	22 (19–24)	0.499
Length of stay (days)^a^	8 (6–12)	10 (7–15)	8 (5–11)	0.124
Chlorthalidone during admission, *n* (%)	23.0 (31.5)	11.0 (52.4)	12.0 (23.1)	**0.015**
Inotropes during admission, *n* (%)	4.0 (5.5)	4 (19)	0	**0.001**
Blood tests				
Glomerular filtration ml/min/1.73 m^2^ ^a^	57.2 (41.9–84.5)	49.3 (28.0–63.2)	69.4 (44.1–86.2)	**0.044**
Urea (mg/dl)^a^	56.0 (41.0–77.5)	74.0 (50.5–80.5)	51 (40–75)	**0.044**
Creatinine (mg/dl)^a^	1.0 (0.8–1.5)	1.3 (0.9–2.1)	1.0 (0.7–1.4)	**0.032**
Sodium (mmol/L)^a^	141 (138–143)	141 (135–143)	141.00 (138.25–143)	0.419
Potassium (mmol/L)^a^	4.3 (4.0–4.9)	4.2 (4.0–4.7)	4.5 (4.0–5.0)	0.294
Chloride (mmol/L)^a^	105 (103–107)	104 (101–106)	105 (103–107)	0.074
Uric acid (g/dl)^a^	8.2 (7.0–9.1)	8.2 (7.0–10.1)	8.2 (6.4–9.1)	0.749
NT-proBNP (pg/ml)^a^	5,691 (2,447–9731)	7583 (3,421–11944)	5,039 (1976–9368)	0.165
Ca 125 (U/ml)^a^	62 (20–128)	57 (9–159)	62 (24–127)	0.606
Albumin (g/dl)^a^	3.9 (3.7–4.1)	3.8 (3.5–4.1)	3.9 (3.7–4.1)	0.213
Cholesterol(mg/dl)^a^	137 (120–161)	129 (114–153)	139 (122–163)	0.434
Aldosterone (ng/dl)^a^	11.5 (7.3–18.0)	19.1 (10.7–40.3)	9.7 (6.8–14.2)	**0.005**
Hemoglobin (g/dl)^a^	12.8 (11.3–14.5)	12.8 (10.9–14.0)	12.8 (11.7–14.6)	0.394

DR, diuretic response; MRA, mineralocorticoids receptor antagonists; SLGT2i, Sodium–glucose cotransporter 2 inhibitors; RAASi, Renin-angiotensin-aldosterone system inhibitors; LVEF, left ventricular ejection fraction; HFmrEF, heart failure with mildly reduced ejection fraction; HFpEF, heart failure with preserved ejection fraction; HFrEF, heart failure with reduced ejection fraction; sPAP, systolic pulmonary artery pressure; TAPSE, tricuspid annular plane systolic excursion; NTproBNP, N-terminal pro-brain natriuretic peptide; CA125, cancer antigen 125.

Bold type: statistically significant values.

a Continous variables are expressed by medians and interquartile range.

During admission, patients with poor diuretic response required more frequently inotropes (19 vs. 0%, *p* = 0.001), and thiazides (52.4 vs. 23.1%, *p* = 0.015).

## Discussion

To date, this is the first study to show the performance of the algorithm proposed by the HF European guidelines for the early assessment of diuretic response in a cohort of patients with acute HF.

This algorithm based on diuresis volume and natriuresis was able to detect up to 29% of patients with insufficient diuretic response who might benefit from enhanced diuretic treatment.

These patients showed some characteristics traditionally described in patients with diuretic resistance in other settings.

### Natriuresis and Diuresis in Acute Heart Failure

Sodium and fluid retention is a hallmark of HF. As effective diuretic response is produced by natriuresis, urinary sodium has emerged as a useful parameter to predict natriuretic response in patients with HF soon after diuretic administration (Verbrugge et al., 2014), which can be measured from a urinary spot sample with good accuracy (Testani et al., 2016). In this line, several studies have reported the usefulness of natriuresis after the first dose of diuretic to predict long-term adverse events (Singh et al., 2014; Honda et al., 2018; Luk et al., 2018; Biegus et al., 2019; Hodson et al., 2019), and two studies have also suggested its usefulness in detecting the development of worsening HF during hospitalization (Collins et al., 2019; Cobo -Marcos et al., 2020).

Although a high diuresis volume following a first intravenous loop diuretic administration is usually associated with good diuretic response and a high urinary sodium (Testani et al., 2016; Singh et al., 2014), some data indicate that in patients with low to medium volume output, spot urinary sodium content offers independent prognostic information (Brinkley et al., 2018). Indeed, in our cohort only 4 patients (5.5%) had both low urinary sodium and a decreased urine output.

Therefore, a spot urine sodium content of *<*50–70 mEq/L after 2 h, and/or an hourly urine output *<*100–150 ml during the first 6 h, provide additional information and could identify patients with an insufficient diuretic response.

### Characteristics of Patients With Insufficient Diuretic Response

The present study confirms findings from previous studies, that a poor response is associated with some features of more advanced disease (Metra et al., 2012; Neuberg et al., 2002; Valente et al., 2014; ter Maaten et al., 2015b; Testani et al., 2014; Voors et al., 2014; ter Maaten et al., 2015a). In our cohort 43% of the patients had a previous HF hospitalization, and the outpatient diuretic dose was high. Besides, compared with patients with an adequate diuretic response, these patients had lower systolic blood pressure at admission, worse glomerular filtration rate, and showed greater neurohormonal activation. It should be noted that variables such as age, left ventricular ejection fraction or natriuretic peptides are not usually associated with the diuretic response in different studies (Metra et al., 2012; Neuberg et al., 2002; Valente et al., 2014; ter Maaten et al., 2015b; Testani et al., 2014; Voors et al., 2014; ter Maaten et al., 2015a). Furthermore, in our cohort no other clinical (Charlson index, Everest score) or echocardiogram features (TAPSE, inferior cava vein) were different between both populations. These data highlight the role of this algorithm in the evaluation of diuretic response in this setting.

Finally, although this study didn’t assess long term events, we showed that patients with a worse diuretic response required diuretic association and inotropes more frequently during admission.

### Feasibility of the ESC Algorithm

At this time, two other studies are evaluating the performance of this diagnostic strategy, the ENACT-HF trial (Rationale and Design of the Efficacy of a Standardized Diuretic Protocol in Acute Heart Failure Study) (Dauw et al., 2021), and the PUSH-HF trial (Natriuresis-guided therapy in acute heart failure: rationale and design of the Pragmatic Urinary Sodium-based treatment algoritHm in Acute Heart) (Maaten et al., 2022).

It should be noted that this novel algorithm involves a more proactive approach and closer monitoring of the diuretic response.

This requires specific training and coordinated and continuos collaboration between the professionals involved in the management of HF patients, especially with emergency department staff, in order to extend the implementation of this diuretic protocol.

### Limitations

Our cohort consisted of 73 patients from one academic institution so the findings may not be generalizable to the wider acute HF population.

In addition, there is a low percentage of patients included (11%) in terms of overall acute HF admissions. Patients with predominantly pulmonary congestion without other congestion signs were not included. Some patients didn’t follow the protocol by decision of the responsible staff. Recruitment was also affected by the COVID-19 pandemic.

## Conclusion

The diagnostic algorithm based on diuresis and natriuresis provided complementary information and was capable of early detection of up to 29% of patients with acute HF from this cohort who presented an insufficient diuretic response.

This finding may help to stratify patients who may benefit from more intense treatment for decongestion during hospital admission.

## Data Availability

The raw data supporting the conclusion of this article will be made available by the authors, without undue reservation.
